# High-resolution Hi-C maps highlight multiscale chromatin architecture reorganization during cold stress in *Brachypodium distachyon*

**DOI:** 10.1186/s12870-023-04269-w

**Published:** 2023-05-16

**Authors:** Xin Zhang, Guangrun Yu, Yan Dai, Hui Zhang, Kai Wang, Jinlei Han

**Affiliations:** School of Life Sciences, Nantong University, Nantong, 226019 China

**Keywords:** Chromatin organization, Hi-C, Histone modification, Cold stress, *Brachypodium distachyon*

## Abstract

**Background:**

The adaptation of plants to cold stress involves changes in gene expression profiles that are associated with epigenetic regulation. Although the three-dimensional (3D) genome architecture is considered an important epigenetic regulator, the role of 3D genome organization in the cold stress response remains unclear.

**Results:**

In this study, we developed high-resolution 3D genomic maps using control and cold-treated leaf tissue of the model plant *Brachypodium distachyon* using Hi-C to determine how cold stress affects the 3D genome architecture. We generated ~ 1.5 kb resolution chromatin interaction maps and showed that cold stress disrupts different levels of chromosome organization, including A/B compartment transition, a reduction in chromatin compartmentalization and the size of topologically associating domains (TADs), and loss of long-range chromatin loops. Integrating RNA-seq information, we identified cold-response genes and revealed that transcription was largely unaffected by the A/B compartment transition. The cold-response genes were predominantly localized in compartment A. In contrast, transcriptional changes are required for TAD reorganization. We demonstrated that dynamic TAD events were associated with H3K27me3 and H3K27ac state alterations. Moreover, a loss of chromatin looping, rather than a gain of looping, coincides with alterations in gene expression, indicating that chromatin loop disruption may play a more important role than loop formation in the cold-stress response.

**Conclusions:**

Our study highlights the multiscale 3D genome reprogramming that occurs during cold stress and expands our knowledge of the mechanisms underlying transcriptional regulation in response to cold stress in plants.

**Supplementary Information:**

The online version contains supplementary material available at 10.1186/s12870-023-04269-w.

## Background

Eukaryotic chromosomes are organized into three-dimensional (3D) configurations inside the cell nucleus and are critical to genome functions [1–3]. Chromosome conformation capture technologies, such as Hi-C, have emerged as powerful tools for chromosome folding analysis and have revealed hierarchical genome organization in both animals and plants. For example, at the megabase scale, the genome can be partitioned into the A and B compartments, which are related to active and inactive chromatin, respectively [4]. Subsequent analyses with a higher resolution (sub-megabase scale) showed that compartments are segregated into topologically associating domains (TADs) [5]. TADs are thought to be the basic functional units of the genome and are conserved across species [5, 6]. With increased resolution (kilobase scale), chromatin loops, which are important for connecting enhancers and promoters that affect gene transcription, can be detected [7, 8]. Recent studies have shown that environmental changes affect 3D chromatin organization in mammalian [9], *Arabidopsis *[10], and rice [11, 12] systems. However, the 3D chromatin behavior in response to abiotic stress in plants remains unclear.

Low temperature is a major environmental limitation that affects plant growth, distribution, and yield [13, 14]. A wide range of plants, including various species of grasses, have evolved sophisticated mechanisms to acquire cold tolerance, such as a process called cold acclimation [13, 15]. During cold acclimation, a variety of genes are reprogrammed, resulting in a series of physiological and metabolic changes that help plants adapt to cold conditions [16]. For example, in *Arabidopsis*, approximately 4 to 20% of the genes in the genome are regulated by cold [17, 18]. Previous studies have elucidated that epigenetic changes, such as DNA methylation [19], histone modifications [20], noncoding RNAs [21], and chromatin accessibility [22, 23], occur during cold acclimation and can play an important role in regulating cold-response gene expression. However, as an integral epigenetic component, 3D chromatin organization and reprogramming during cold stress remain to be elucidated. Thus, investigating the 3D organization of chromatin is critical for uncovering the mechanism behind plant adaptation to cold and for the further development of plants with increased cold tolerance.

*Brachypodium distachyon* is native to the Mediterranean region and has been established as a model for cold-induced responses in temperate cereals [24]. Here, we examine the dynamics of 3D chromatin organization during cold stress in *Brachypodium distachyon* by using Hi-C analysis. Our results revealed that cold stress has a widespread impact on chromatin organization, including compartments, TADs, and long-range interactions. By integrating Hi-C, RNA-seq, histone ChIP-seq, and DNase-seq, we found that changes in chromatin organization were associated with epigenetic states and gene expression changes. These results provide valuable information for deciphering the role of the 3D chromatin landscape in the cold-stress response.

## Results

### Global chromatin architecture is reorganized during cold stress

**Fig. 1:**
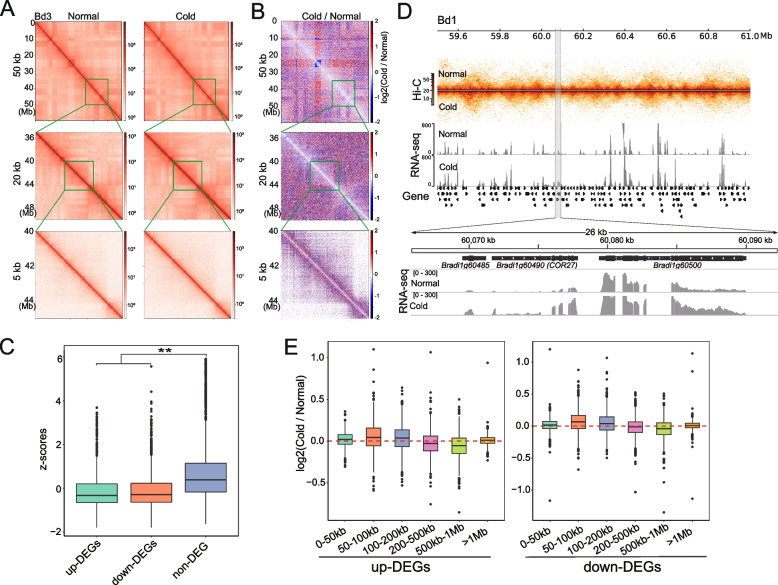
Comparison of chromatin interaction profiles between normal and cold-treated Bd21. **A** Multiresolution Hi-C contact heatmaps for normal and cold-treated Bd21. Normalized Hi-C matrices were constructed at resolutions of 50 kb, 20 kb, and 5 kb. **B** Hi-C heatmaps of relative contact differences between normal and cold-treated Bd21. The relative difference is calculated as the log2 ratios of the Hi-C matrices of the cold-treated and normal samples. In the heatmap, Hi-C interactions that become stronger under cold conditions compared with normal conditions are in white to red, whereas Hi-C interactions that become weaker under cold conditions compared with normal conditions are in white to blue. Hi-C interactions that do not change under cold compared with normal conditions are in white. **C** Boxplots of CHESS z-scores for genomic windows with and without genes differentially expressed between cold-treated and normal Bd21. Low values indicate greater differences in chromatin conformation. The Wilcoxon test was used to analyse statistical significance. ‘**’ represents a *P* value < 0.01. **D** Hi-C heatmaps and RNA-seq tracks surrounding the *Bradi1g60490* (*COR27*) locus (grey region). **E** Boxplots of changes in relative contact frequencies between gene promoters and their distal regions under cold stress

We previously reported that cold stress induces transcriptional changes in thousands of genes in *Brachypodium distachyon* (inbred line Bd21) [23]. To uncover the role of 3D chromatin architecture in transcriptional responses during cold stress, we performed Hi-C experiments on Bd21 under normal and cold-treated (24 h at 4 ℃) conditions with two biological replicates, generating a total of 1.2 billion sequencing read pairs (Table S1). After alignment to the reference genome and filtering, we obtained over 205 and 247 million valid Hi-C reads for normal and cold-treated samples, respectively. Quality assessment showed that the Hi-C library was of high quality (QuASAR-QC scores 0.025–0.042) and had high reproducibility between replicates (stratum-adjusted correlation coefficient (SCC) > 0.95) (Fig. S1). Thus, we merged the replicates to obtain a maximum resolution of the Hi-C matrix of approximately 1.5 kb for each condition for subsequent analyses (Table S1). At different resolutions, Hi-C revealed hierarchical, higher-order chromatin structures (Fig. 1A).

First, we investigated the difference in the global Hi-C contact maps between the normal and cold-treated samples. We noticed prominent changes in the interaction frequency, with increased short-range interactions and diminished mid/long-range interactions under cold stress (Fig. 1B). This dual effect was also evident in the analysis of contact probability, which was dependent on the genomic distance (Fig. S2). To determine whether changes in chromatin conformation were accompanied by changes in transcription, we classified the genes as upregulated and downregulated under cold stress using the RNA-seq data. We identified 1,439 upregulated genes and 1,831 downregulated genes (Fig. S3). Examination of Comparison of Hi-C Experiments using Structural Similarity (CHESS) scores in windows containing up- or downregulated genes revealed a tight association between differential gene expression and differential chromatin structure (*P* < 0.01, Wilcoxon test) (Fig. 1C). For instance, closer examination of a locus harbouring a classical cold-response gene, *Bradi1g60490* (*COR27*), showed 2.8-fold transcriptional activation, and an obvious change in the composition of the Hi-C contact heatmaps (Fig. 1D). Interestingly, we observed that both upregulated and downregulated genes exhibited an increase in short-range interactions, particularly in the 50 kb-100 kb range (Fig. 1E). In contrast, we found a reduction in mid/long-range interactions, especially in the 500 kb-1 Mb range. These findings suggest that increased short-range interactions and decreased mid/long-range interactions play a dual role in regulating gene expression, which may either upregulate or downregulate gene expression. This dual role highlights the complexity of 3D genome regulation in gene expression. Together, cold stress perturbs global chromatin architecture, which may facilitate concomitant shifts in transcriptional activity.

### Cold stress results in a reduction in chromatin compartmentalization

**Fig. 2:**
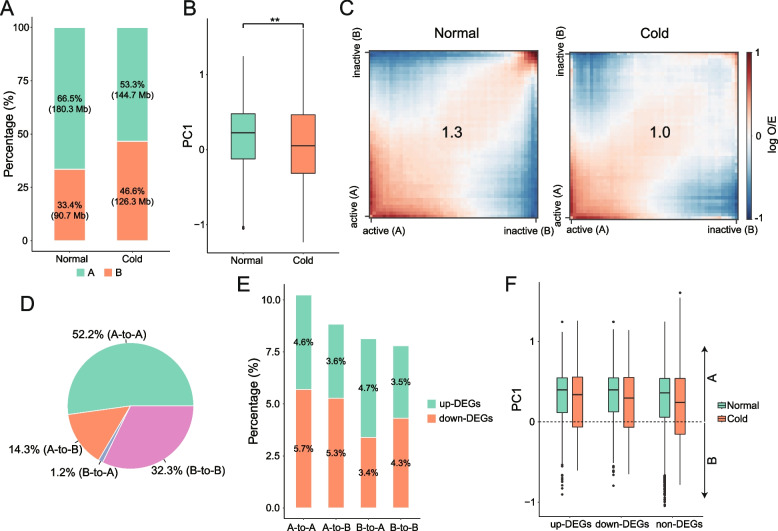
The A/B compartment switches and compartmentalization strength decreases during cold stress in Bd21. **A** The proportions and lengths of A/B compartments in the Bd21 genome. **B** Comparison of PC1 values obtained from the Hi-C data for normal and cold-treated Bd21. A positive value indicates the A compartment, and a negative value indicates the B compartment. The Wilcoxon test was used to analyse statistical significance. ‘**’ represents a *P* value < 0.01. **C** Saddle plot of Hi-C data. Saddle plots were constructed using the FAN-C compartments function. Interactions between A compartments are in the bottom left, and interactions between B compartments are in the top right. The numbers at the centre of the heatmaps indicate compartment strength, calculated as: the natural logarithm of (AA * BB) / AB^2. **D** The percentage of stable (A-to-A or B-to-B) or switched compartments (A-to-B or B-to-A) between normal and cold-treated Bd21 in the genome. **E** The percentage of DEGs in stable (A-to-A or B-to-B) or switched (A-to-B or B-to-A) compartments. **F** Boxplots showing PC1 values of compartments containing different classes of genes under both normal and cold stress conditions. A positive value indicates the A compartment, and a negative value indicates the B compartment

Hi-C revealed the presence of A and B compartments corresponding to active and inactive transcriptional regions, respectively, in mammalian and plant genomes [4, 8, 25]. We then investigated the effect of cold stress on genomic compartments, denoted by PC1 values obtained through principal component analysis (PCA) of the Hi-C contact matrices (Table S2). As expected, the A compartments showed significantly higher gene density and transcription levels than the B compartments (*P* < 0.01, Wilcoxon test) (Fig. S4). Compared with the normal sample, we found that cold stress promoted an increase in the number of genomic sequences defined as B compartments (Fig. 2A) and lower PC1 values overall (*P* < 0.01, Wilcoxon test) (Fig. 2B). In total, 66.5% (180.3 Mb) and 33.4% (90.7 Mb) of the genomic regions were identified as A and B compartments, respectively, in normal samples, whereas 53.3% (144.7 Mb) of A and 46.6% (126.3 Mb) of B compartments were identified in cold-treated samples (Fig. 2A). Furthermore, we assessed the compartment strength using the generated saddle plots. Notably, for all samples, compartments of the same type (A-A, B-B) had a higher frequency of contacts than compartments of different types (A-B) (Fig. 2C), consistent with previous observations [4, 26]. However, we determined a reduction in compartmental interactions, especially B-B interactions, indicative of weakened compartmentalization upon cold stress (compartment strength 1.3 in normal vs. 1.0 in cold-treated sample) (Fig. 2C).

To explore the impact of compartment changes on gene expression, we focused on genomic regions exhibiting dynamic switching of A/B compartment status upon cold stress. We found that 15.5% (42.1 Mb) of the genome switched compartment states, with more A compartments switching to B compartments (14.3%, 38.9 Mb) than B to A compartments (1.2%, 3.2 Mb), upon cold stress (Fig. 2D). There were 5,294 and 295 genes that resided in the genomic regions that switched from compartment A to B and from B to A under cold stress, respectively. If changes in the compartment were accompanied by changes in transcription, compartment switching regions would be enriched for differentially expressed genes (DEGs). However, only ~ 9% of the genes were DEGs in compartment switching regions, with no major difference in compartments with stable status (Fig. 2E). Both cold stress up- and downregulated genes were predominantly localized in the A compartment in both the normal and cold-treated samples (Fig. 2F). The inconsistent relationship indicates the uncoupling of compartment switching and gene expression, similar to the results in *Drosophila* and peanut [27, 28].

### Cold stress affects TAD structure, leading to transcriptome changes

**Fig. 3:**
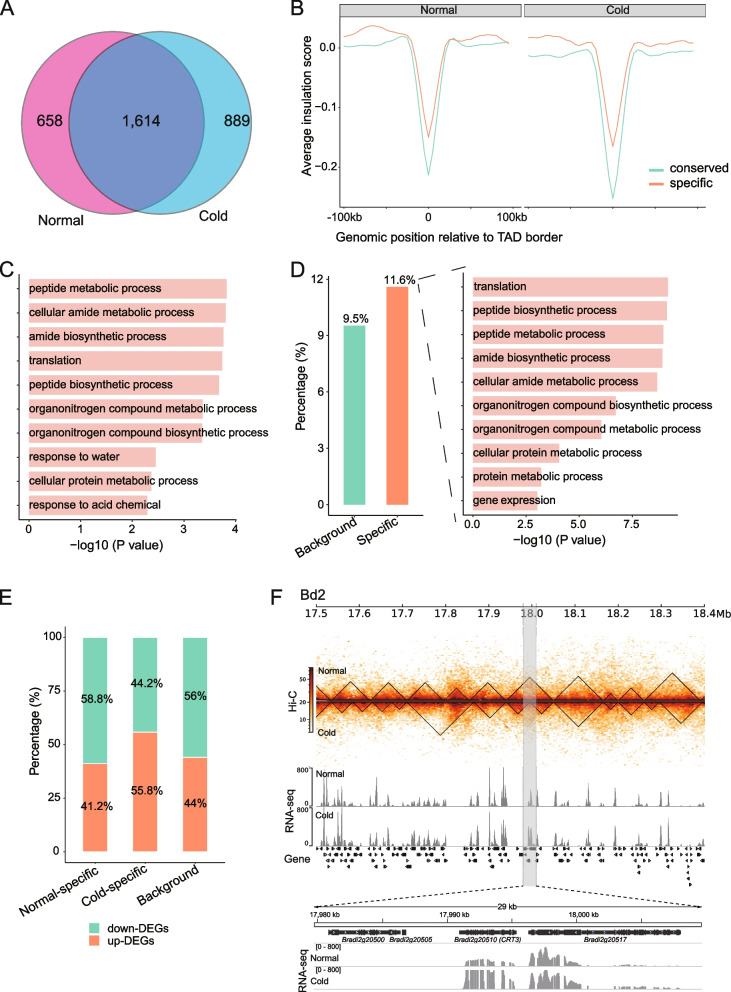
TAD dynamics during cold stress in Bd21. **A** Overlap of TAD boundaries in normal and cold-treated Bd21. Consensus borders were defined by allowing a shift of two bins (10 kb) in each direction between the two conditions. **B** Comparison of insulation scores between the conserved and specific boundaries in each sample. **C** Gene Ontology (GO) biological process analysis of genes in specific TAD boundaries. The top ten enriched GO biological processes are indicated. **D** The proportion of differentially expressed genes in specific TAD boundaries. The overall prevalence of DEGs is the control group (background). GO enrichment analysis of differentially expressed genes in specific TAD boundaries was performed. The top ten enriched GO biological processes are indicated. **E** Relative proportion of upregulated and downregulated DEGs in normal- and cold-specific TAD boundaries, respectively. The overall DEGs are the control group (background). **F** An example of a TAD boundary, which is gained in cold stress, can be observed in a region on chromosome 2 (grey region). TADs identified as regions of high chromatin interactions are outlined by black triangles

At a local level, Hi-C can be used to characterize TADs, which are considered important structural and functional units of the 3D genome [11, 29]. To investigate whether cold stress affects TAD structures, we defined TADs at 5 kb resolution in normal and cold-treated samples using insulation score (IS) algorithms (see Methods). A total of 2,267 TADs were identified in the normal sample and 2,499 TADs in the cold-treated sample (Table S3). As expected, TAD boundaries were enriched for genes and had a lower IS than flanks (Fig. S5), indicating the reliability of the identified TADs. However, we found that cold stress strengthened insulation at TAD boundaries (Fig. S5), suggesting that chromatin interactions decreased across TAD boundaries. We then performed TAD comparisons and found that the majority of TAD boundaries (1,614, 71.0%) present in the normal sample were conserved in the cold-treated sample, and that only 889 and 658 TAD boundaries were gained or lost as cold- or normal-specific boundaries (Fig. 3A). Comparison of the IS of conserved and cold/normal-specific TAD boundaries showed that conserved boundaries have stronger insulation (Fig. 3B).

Subsequently, we investigated whether TAD reconstruction contributes to dynamic gene expression during cold stress. We examined the genes at dynamic TAD boundaries. The normal- and cold-specific TAD boundaries harboured 638 and 846 genes, respectively, which were related to several protein translation- and environmental response-related processes, such as peptide metabolic and biosynthetic processes, amide metabolic and biosynthetic processes, and response to water or chemical substances (Fig. 3C). Notably, we found that DEGs were enriched within these specific boundaries: 11.6% (172/1484) of genes within dynamic boundaries, including 68 genes within normal-specific TAD boundaries and 104 genes within cold-specific TAD boundaries, were differentially expressed genes compared to the overall prevalence of DEGs (9.5%, percentage of DEGs relative to total genes) (*P* < 0.01, Fisher’s exact test) (Fig. 3D). GO enrichment analysis showed that these genes are involved in translation, peptide and amide metabolic and biosynthetic processes (Fig. 3D). In addition, we demonstrated that boundary gain was associated with upregulated gene expression (1.3-fold up-DEGs vs. down-DEGs), whereas boundary loss was associated with downregulated gene expression (1.4-fold down-DEGs vs. up-DEGs) (Fig. 3E). For example, *Bradi2g20510* (*CRT3*) encodes calreticulin, which plays a role in calcium ion homeostasis and tolerance to abiotic stress [30]. When it gained a TAD boundary, its expression increased under cold stress conditions (Fig. 3F). Although we observed a correlation between boundary gain/loss and up/downregulated genes, we cannot conclude that boundary changes are a prerequisite for differential gene expression. Further experimental validation and analysis are necessary to confirm this relationship. Overall, the reconstruction of TADs during cold stress in Bd21 affected gene expression.

### Changes in TADs associated with epigenetic state alterations during cold stress

**Fig. 4:**
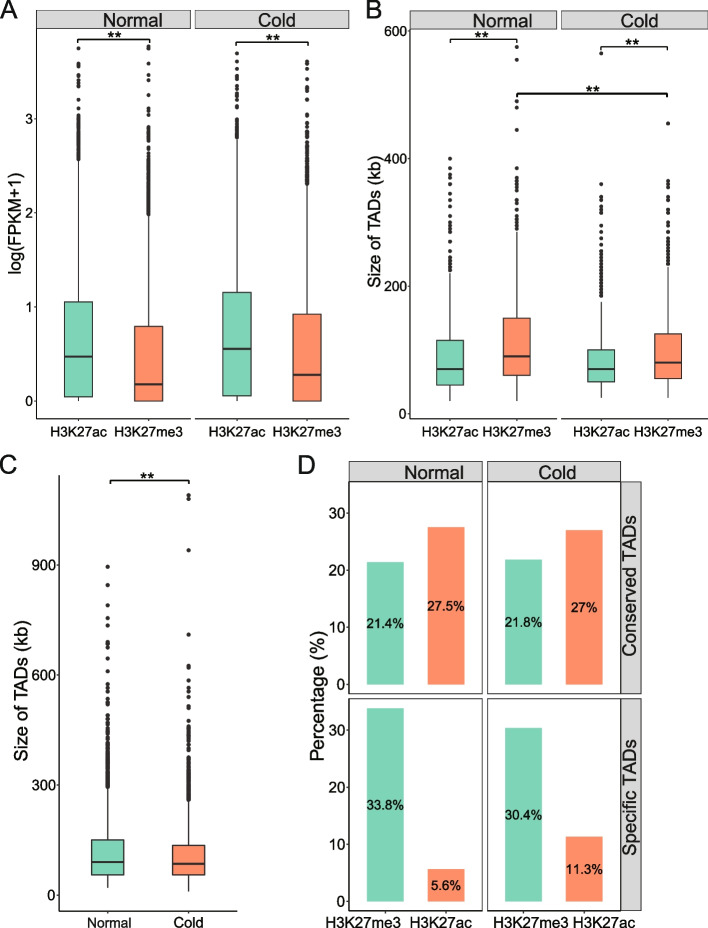
Identification of histone mark-enriched TADs. **A** Expression levels of genes located in each histone mark-enriched TAD. **B** The size of each histone mark-enriched TAD across the two conditions. **C** The size of TADs in normal and cold-treated Bd21. The Wilcoxon test was used to analyse statistical significance in (**A**)-(**C**). ‘**’ represents a *P* value < 0.01. **D** Percentage of histone mark-enriched TADs among the conserved, normal-specific, and cold-specific TADs

To explore the factors driving TAD alterations during cold stress, we characterized TADs by performing chromatin immunoprecipitation and sequencing (ChIP-seq) of two histone modifications (H3K27ac and H3K27me3) in normal and cold-treated samples. Similar to the study of other plants [31], the active histone modification H3K27ac was enriched in active genes at the transcription start sites (TSSs) (Fig. S6). In contrast, the repressive marker H3K27me3 was enriched in inactive genes across the gene body (Fig. S6). Thus, we used H3K27ac to annotate transcriptionally active TADs, and H3K27me3 to annotate repressed TADs. As expected, the average gene expression levels of H3K27me3-enriched TADs were lower than those of H3K27ac-enriched TADs (*P* < 0.01, Wilcoxon test) (Fig. 4A). The H3K27me3-enriched TADs were also larger in size (*P* < 0.01, Wilcoxon test) (Fig. 4B), which is consistent with the fact that H3K27me3 covers large heterochromatic regions [32]. Interestingly, we found that the size of H3K27me3-enriched TADs decreased significantly during cold stress (*P* < 0.01, Wilcoxon test) (Fig. 4B), similar to the overall trend of smaller TADs in cold-treated samples (*P* < 0.01, Wilcoxon test) (Fig. 4C). However, at H3K27ac-enriched TADs, the size of the TADs was similar in normal and cold-treated samples (Fig. 4B). This result suggests that TAD splitting can occur during cold stress and that H3K27me3 may mediate the formation of new TADs. Next, we specifically examined the epigenetic states within the sets of conserved, normal-specific, and cold-specific TADs. We found that normal-specific TADs had a higher percentage of H3K27me3-enriched TADs than cold-specific or conserved TADs (Fig. 4D). In contrast, the percentage of H3K27ac-enriched TADs was lower in normal-specific TADs, indicating that inactive TADs may switch to active TADs during cold stress. This finding further supports the notion that TAD reconstruction may be coupled with epigenetic alterations during cold stress.

### Cold stress disturbs the long-range chromatin loop

**Fig. 5:**
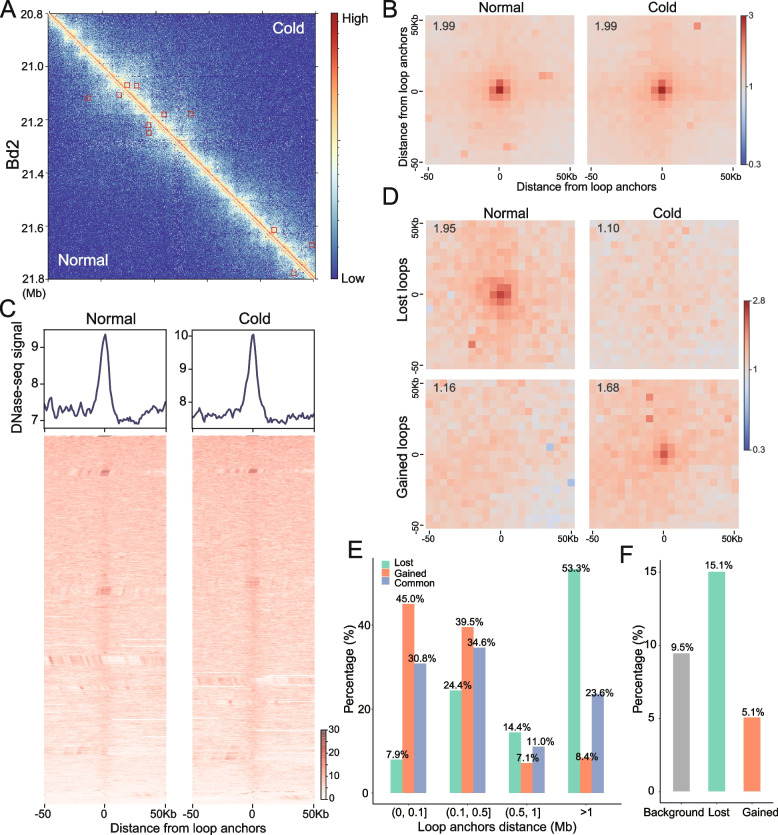
Detection of differential looping events during cold stress. **A** Hi-C contact matrix (2-kb resolution) depicting normalized contact frequencies for normal (lower left) and cold-treated (upper right) Bd21. The chromatin loop is highlighted by red boxes. **B** APA plots showing aggregated signals across all loops in normal and cold-treated Bd21. The APA value is displayed on the top left. **C** Heatmaps and average profiles showing the normalized DNase-seq tag density located within 50 kb upstream and downstream of the centre of the loop anchors in normal and cold-treated Bd21. **D** APA plots of loops lost or gained during cold stress. Individual plots for each condition are shown. The APA value is displayed on the top left. **E** Distance distributions of the lost, gained, and common loops. **F** The proportion of differentially expressed genes whose promoters overlapped with the anchors of lost or gained loops during cold stress. The overall prevalence of DEGs is the control group (background)

Chromatin loops connect distal cis-regulatory elements (CREs), such as enhancers to their target genes, which are thought to play an important role in gene expression regulation [33]. We identified significant chromatin loops in normal and cold-treated samples to understand the dynamics of the regulatory loops during cold stress. A total of 2,274 loops were identified in the normal sample, and 1,887 loops were identified in the cold-treated sample (Fig. 5A, Table S4). The accuracy of the resulting loop calls was supported by high-scoring aggregate peak analysis (APA) plots, which were used to assess the aggregate strength of the loops (Fig. 5B). To explore the regulatory nature of DNA looping, we intersected loop calls with our previous DNase-seq data [23]. We found enrichment of DNase-seq signals in loop anchor regions (Fig. 5C). Additionally, the majority (~ 80%) of loop anchors overlapped with DNase-seq peaks (DHSs), which were expected to encompass CREs (Fig. S7). The loop anchor enrichment at DHSs was statistically significant compared with random genomic regions with the same size of loop anchors (empirical *P* value < 0.001), supporting a regulatory role of these loops.

Next, we determined the dynamic looping events following cold stress. Using a method similar to calling peaks with control data [34], we identified 238 gained loops and 291 lost loops under cold stress (cLoop2, *P* < 0.01; see Methods) (Table S5). The APA plots illustrated that the dynamic loops showed a significant difference in the contact frequencies before and after cold treatment (Fig. 5D), suggesting that our stringent approach identified the differential loops during cold stress. We then compared gained and lost loops across different ranges of genomic distance (Fig. 5E). Long-range loops (> 1 Mb) showed the most dramatic difference: lost loops were present 6.3 times more often than gained loops (53.3% versus 8.4%; *P* < 0.01, Fisher’s exact test) and were 2.3 times more abundant than common loops in both samples (*P* < 0.01, Fisher’s exact test). Therefore, we conclude that adaptation to cold stress correlates with widespread disruption of long-range loops.

Our previous work reported cold-response DHSs, including 1706 gained DHSs and 267 lost DHSs in Bd21 [23]. To investigate the influence of changes in chromatin accessibility on loop dynamics, we intersected different DHSs with different loops. We found that only 10 lost loop anchors overlapped with lost DHSs, and 34 gained loop anchors overlapped with gained DHSs. The lack of overlap suggests that changes in chromatin accessibility are unlikely to be involved in loop dynamics. We also examined the enrichment of H3K27ac and H3K27me3 signals in the differential loop anchor regions and found no significant change in enrichment during cold stress (Fig. S8). These findings imply that cold stress rewires the DNA loops in an epigenetically independent manner. Furthermore, we considered how genes whose promoters (defined as 2 kb regions upstream of the transcription start sites) overlapped anchors of these two loop sets changed during cold stress. The lost and gained loops overlapped in 73 and 98 genes, respectively. Compared with the genome background (9.5%, percentage of DEGs relative to total genes), DEGs were 1.6-fold overrepresented in lost loops (15.1%, 11/73) but not in gained loops (5.1%, 5/98) (Fig. 5F). Overall, these findings indicated that loop disruption, as opposed to loop formation, may play a broader role in the Bd21 cold response.

## Discussion

The 3D genome structure is a critical regulator of multiple biological processes in eukaryotic organisms. However, the role of the 3D genome structure in plant cold acclimation is unclear. Here, we used the Hi-C approach to determine the 3D genome response to cold stress and how such changes might correlate with the transcriptional responses in *Brachypodium distachyon*. These results provide new insights into the responses and regulation of plants under cold stress conditions.

A comparison of the Hi-C maps revealed that the chromatin folding patterns after one day of cold treatment were dramatically different from those of the control plants. We found that cold stress induced short-range chromatin interactions but weakened mid/long-range chromatin interactions (Fig. 1B, Fig. S2), similar to previous observations in rice in response to cold stress [12]. Interestingly, in *Arabidopsis*, it has been reported that heat stress induces long-range interactions but weakens short-range chromatin interactions [35]. These results suggest that cold and heat exert opposite effects on chromatin architecture, which might be intuitive as basic thermodynamics indicate that molecules expand with heat and contract with cold. Furthermore, we analysed the chromatin compartments. Consistent with previous studies [10, 35], transition of compartments (A-to-B or B-to-A) was observed in stress-treated plants (Fig. 2D). Notably, cold-response genes were overrepresented in nontransitional compartments (for example, A compartments) (Fig. 2F), suggesting that the A compartment was more easily accessible for transcriptional reprogramming after cold stress. Meanwhile, we found that cold treatment weakened compartmentalization (Fig. 2C), which supports the previous proposition that cold reduces spatial constraints on chromosomal compartments [12].

At the local level, we identified TADs that tended to be smaller and more numerous in the cold-treated plants. We called more than one thousand specific TAD boundaries between normal and cold-treated plants (Fig. 3A). Chromatin identified as TAD boundaries only in cold-treated plants had different Hi-C patterns from those of the control plants, and vice versa. Although over 1400 genes were located within these specific boundaries, only 172 (11.6%) of these genes showed significantly altered expression during cold stress (Fig. 3D). Although this ratio is higher than the overall prevalence of DEGs, supporting the model that chromatin structural changes can contribute to gene regulation [36], it also highlights that chromatin structure is one of many factors involved in transcriptional control. Notably, by combining histone modification data, we observed different distribution patterns of H3K27ac and H3K27me3 in the TADs. More than 30% of the condition-specific TADs were H3K27me3-enriched (Fig. 4D). In contrast, only a small percentage (5.6-11.3%) of these TADs were H3K27ac-enriched. These findings validate and expand our knowledge of the correlation between TADs and histone modifications [36, 37].

Our high-resolution Hi-C maps (~ 1.5 kb) also allowed the identification of chromatin loops. As expected, the loop anchors were highly enriched at DHSs (e.g., CREs) (Fig. 5C), indicating that the experimental results were robust. We performed quantitative comparisons of contact frequency between normal and cold-treated plants to identify differences in chromatin looping and integrated these differences with differentially expressed genes to examine their functional relevance. Our findings suggest that loss of looping coincides with alterations in gene expression (Fig. 5F). In contrast, only a small fraction (5.1%) of the genes showed differential expression accompanying gain of looping. Previous studies in macrophages revealed that loop disruption inhibits the activation of proinflammatory transcription [38], suggesting that loop loss plays a role in regulating gene expression. However, other studies have shown that loss of cohesin eliminates most chromatin interaction loops in HCT-116 (a human colorectal carcinoma cell line) but does not substantially alter gene transcription [39]. In addition, in some cases, the gained loops can play a role in gene activation [36], and events beyond the loss of loops are required to change gene expression [28]. Although it is difficult to fully reconcile these findings, one possible explanation that loops alone may not be sufficient for transcriptional regulation may account for the disparity. Indeed, accumulating evidence has shown that gene transcription is a complex process regulated by a myriad of factors, including 3D chromatin architecture, chromatin accessibility, DNA methylation, noncoding RNAs and modification of chromatin-associated proteins [40–43]. Further functional studies are required to elucidate the exact role of chromatin looping in gene activation and repression.

In summary, we assessed the impact of cold stress on 3D chromatin maps and their implications in *Brachypodium distachyon*. We reveal that cold stress-induced genome reorganization, involving changes in A/B compartments, TADs, and chromatin loops, can account for 20.2% (661/3270) of DEGs, which are involved in translation, peptide biosynthesis and metabolic processes, and amide biosynthesis and metabolic processes (Fig. S9). The Hi-C maps provide a valuable resource for the cold acclimation field and for further studies on the relationship between chromatin interactions and transcriptional regulation.

## Conclusions

In summary, we performed a comprehensive study of the cold stress response in *Brachypodium distachyon* by compiling Hi-C, RNA-seq, histone ChIP-seq and DNase-seq data. Comparative analysis revealed that cold stress disrupts different levels of chromosome organization, which are associated with transcription and histone modification state alterations. Moreover, we also found that loss of chromatin looping, rather than gain of looping, coincided with alterations in gene expression in the cold stress response. The findings emphasize the importance of chromatin structure reprogramming in cold acclimation and advance our understanding of the regulatory architecture of plants in response to cold.

## Materials and methods

### Plant materials and growth conditions

The plant materials were cultured as previously described [23]. Briefly, seeds of *Brachypodium distachyon* (inbred line Bd21) were germinated on wet filter paper at 30 °C on Petri plates. The seedlings were transferred to potting soil and grown under environmentally controlled greenhouse conditions (16 h/8 h of light/dark, 22 °C light/20 °C dark). After 15 d, half of the plants were directly transferred to an incubator set at 4 °C treatment for 24 h (16 h/8 h light/dark). Leaf tissues from the control and cold-treated plants were collected and immediately frozen in liquid nitrogen for further analysis.

### Hi-C library construction and sequencing

The Hi-C library construction was performed at Frasergen Co., Ltd (Wuhan, China). Approximately 2 g of leaf tissue from two replicates was ground to a powder in liquid nitrogen for the Hi-C experiment. The procedure for the Hi-C experiment, including DNA cross-linking, chromatin digestion (restriction enzyme: *Mbo*I), marking of DNA ends with biotin, in situ ligation of proximal ends, reversal of crosslinking, and DNA purification, was performed following a previously published protocol [44]. The library was sequenced with 150 bp paired-end reads on an Illumina HiSeq platform.

### Hi-C data processing

The raw reads generated from Hi-C were quality filtered and trimmed using trim_galore v.0.6.7 (https://www.bioinformatics.babraham.ac.uk/projects/trim_galore/). Each mate of the cleaned read pairs was aligned to the reference genome using BWA-MEM algorithm in the BWA v.0.7.17-r1188 software (http://bio-bwa.sourceforge.net) with parameters “-A1 -B4 -E50 -L0”. The genome sequence and annotation files for *B. distachyon* (v.3.1) were downloaded from Phytozome 12.1 (http://phytozome.jgi.doe.gov/pz/portal.html). The hicBuildMatrix, hicNormalize, hicCorrectMatrix and hicCompareMatrices from HiCExplorer v.3.7.2 [45] were used to construct 2 kb, 5 kb, 20 kb, and 50 kb Hi-C matrices, normalized for sequencing depth, perform Knight-Ruiz (KR) correction, and generate log2 ratios of interaction frequency matrices between normal and cold-treated samples, respectively. The quality and reproducibility of the Hi-C data were assessed using QuASAR-QC and stratum-adjusted correlation coefficient (SCC) scores calculated using 3D Chromatin-ReplicateQC package [46]. The Hi-C resolution was estimated using HiCRes v.1.1 [47]. In addition, we applied CHESS v.0.3.7 [48] to 5 kb resolution Hi-C matrices using windows of 500 kb and a step size of 5 kb to produce Hi-C similarity scores for Hi-C comparisons of normal and cold-treated samples. Hi-C contact maps were plotted using hicPlotMatrix of HiCExplorer and pyGenomeTracks v.3.7 [49].

### ChIP-seq and data analysis

The ChIP-seq assay was performed according to a published protocol [31]. Two commercial antibodies, H3K27me3 (Sigma, Cat. #07–449) and H3K27ac (Sigma, Cat. #07–360), were used for immunoprecipitation. The ChIP DNAs were ligated with Illumina sequencing adaptors and sequenced using the Illumina HiSeq platform to produce 150 bp paired-end reads. The raw reads generated from ChIP-seq were quality filtered and trimmed using trim_galore. Cleaned reads were mapped to the *B. distachyon* genome using Bowtie2 v.2.2.5 [50] with default parameters. Mapped reads were filtered using SAMtools v.1.9 [51] to retain only correctly read pairs with a mapping quality score ≥ 10 for further analysis. Peak calling was performed using MACS2 v.2.1.4 [52] with the parameters “-f BAMPE –broad -g 2.7e8 –nomodel”.

### Identification of A and B compartments

The principal component analysis (PCA) method in the cooltools package (https://github.com/open2c/cooltools) was applied to identify A and B compartments with a bin resolution of 50 kb. We used H3K27ac ChIP-seq data to determine the orientation of eigenvectors (PC1). The A compartment bins were assigned with positive eigenvector values, and the B compartment bins were assigned with negative eigenvector values. We analysed the strength of the compartment by generating saddle plots using the compartments command provided in FAN-C v.0.9.24 [53] with parameters “-p 2 4 6 … 98 100 –compartment-strength”.

### Identification of TADs

TADs and boundaries were identified by the hicFindTADs of HiCExplorer software at 5 kb resolution with parameters “–minDepth 20,000 –maxDepth 300,000 –step 5000 –correctForMultipleTesting fdr –thresholdComparisons 0.01 –delta 0.01”. The TAD insulation scores were generated when identifying TAD boundaries, which were used to identify the degree of separation between the left and right regions of each Hi-C matrix bin. The negative insulation score corresponds to the boundary strength. Histone mark-enriched TADs were defined as TADs intersecting with corresponding ChIP-seq peaks and having the top 25% ChIP-seq signals. Intersections were performed using BEDTools v.2.26.0 [54]. The differential TADs between normal and cold-treated samples were detected by the hicDifferentialTAD of HiCExplorer at 5 kb resolution with parameters “-p 0.05 -mr all”.

### Identification of chromatin loops

Loop calling was carried out using cLoops2 v.0.0.3 [34] with parameters “-eps 2000,5000,10,000 -minPts 20,50 -w -j -hic”. To identify the loops that were differentially enriched under normal and cold-treated conditions, Hi-C reads were sampled to same depth using the cLoops2 samplePETs module, and differentially loops were called using the cLoops2 callDiffLoops module. Aggregated plots at loops were generated using Coolpup.py v.1.0.0 [55] at 5 kb resolution.

### DNase-seq and RNA-seq data analysis

DNase-seq and RNA-seq data of normal and cold-treated *B. distachyon* leaf tissues were obtained from our previous publication (European Nucleotide Archive (ENA) accession number: PRJEB32944) [23]. For DNase-seq, clean reads were mapped to the genome with Bowtie2 and processed to keep those with a mapping quality score ≥ 10. For RNA-seq, clean reads were mapped to the genome with HISAT2 v.2.1.0 (https://ccb.jhu.edu/software/hisat2/index.shtml) and processed to keep only correctly read pairs with a mapping quality score ≥ 10. Expression values were calculated in terms of FPKM (fragments per kilobase of transcript per million fragments mapped) for each gene with Cufflinks v.2.2.1 [56]. FeatureCounts v.2.0.1 [57] was used to calculate the read counts per gene and differential expression analysis was performed using DEseq2 (adjusted *p*-value < 0.05, |log2fold change|> 1) [58]. GO (Gene Ontology) enrichment analysis was performed using an online resource (www.omicshare.com/tools) with default instructions.

## Supplementary Information

**Additional file 1:** **Figure S1.** Quality measures for the Hi-C maps. **Figure S2.** Interaction decay exponents along with genomic distance at 50-kb resolution of normal (blue) and cold-treated (red) Bd21. **Figure S3.** Volcano plots of differentially expressed genes (DEGs) in Cold vs. Normal samples. **Figure S4.** Illustration of gene density and gene expression (FPKM value) of A compartments and B compartments in the normal and the cold-treated Bd21, respectively. **Figure S5.** Genes enrichment and insulation score profiles around TAD boundaries at the two conditions. **Figure S6.** Distribution of histone modifications (H3K27ac and H3K27me3) surrounding genes in the normal and the cold-treated Bd21, respectively. **Figure S7.** The proportion of loop anchors overlapped with DNase-seq peaks (DHSs). **Figure S8.** Line plots showing the normalized tag intensity of H3K27ac (left) or H3K27me3 (right) data from normal or cold treated Bd21 at lost or gained loop anchors. **Figure S9.** GO biological process analysis of DEGs associated with genome reorganization. The top ten enriched GO biological processes are indicated.

**Additional file 2:** **Table S1.** Hi-C data summary.

**Additional file 3:** **Table S2.** A/B compartments identified at 50 kb resolution.

**Additional file 4:** **Table S3.** TADs identified at 5 kb resolution.

**Additional file 5:** **Table S4.** Chromatin loops identified in normal and cold-treated plants.

**Additional file 6:** **Table S5.** Differential loops were identified between normal and cold-treated plants.

## Data Availability

The Hi-C and ChIP-seq data described in this work have been deposited to the Genome Sequence Archive (GSA) database (http://gsa.big.ac.cn/) under the accession number CRA008943. The data can be viewed through the reviewer link (https://ngdc.cncb.ac.cn/gsa/s/WwCx1K7s).
