# Supplementary material for: High-resolution Hi-C maps highlight multiscale chromatin architecture reorganization during cold stress in Brachypodium distachyon
Source: BMC Plant Biol. 2023 May 16;23:260. doi: 10.1186/s12870-023-04269-w (PMC10186695; doi:10.1186/s12870-023-04269-w)
Supplement: Supplementary file 1 — Additional file 1: Figure S1. Quality measures for the Hi-C maps. Figure S2. Interaction decay exponents along with genomic distance at 50-kb resolution of normal (blue) and cold-treated (red) Bd21. Figure S3. Volcano plots of differentially expressed genes (DEGs) in Cold vs. Normal samples. Figure S4. Illustration of gene density and gene expression (FPKM value) of A compartments and B compartments in the normal and the cold-treated Bd21, respectively. Figure S5. Genes enrichment and insulation score profiles around TAD boundaries at the two conditions. Figure S6. Distribution of histone modifications (H3K27ac and H3K27me3) surrounding genes in the normal and the cold-treated Bd21, respectively. Figure S7. The proportion of loop anchors overlapped with DNase-seq peaks (DHSs). Figure S8. Line plots showing the normalized tag intensity of H3K27ac (left) or H3K27me3 (right) data from normal or cold treated Bd21 at lost or gained loop anchors. Figure S9. GO biological process analysis of DEGs associated with genome reorganization. The top ten enriched GO biological processes are indicated. [file 12870_2023_4269_MOESM1_ESM.pdf]

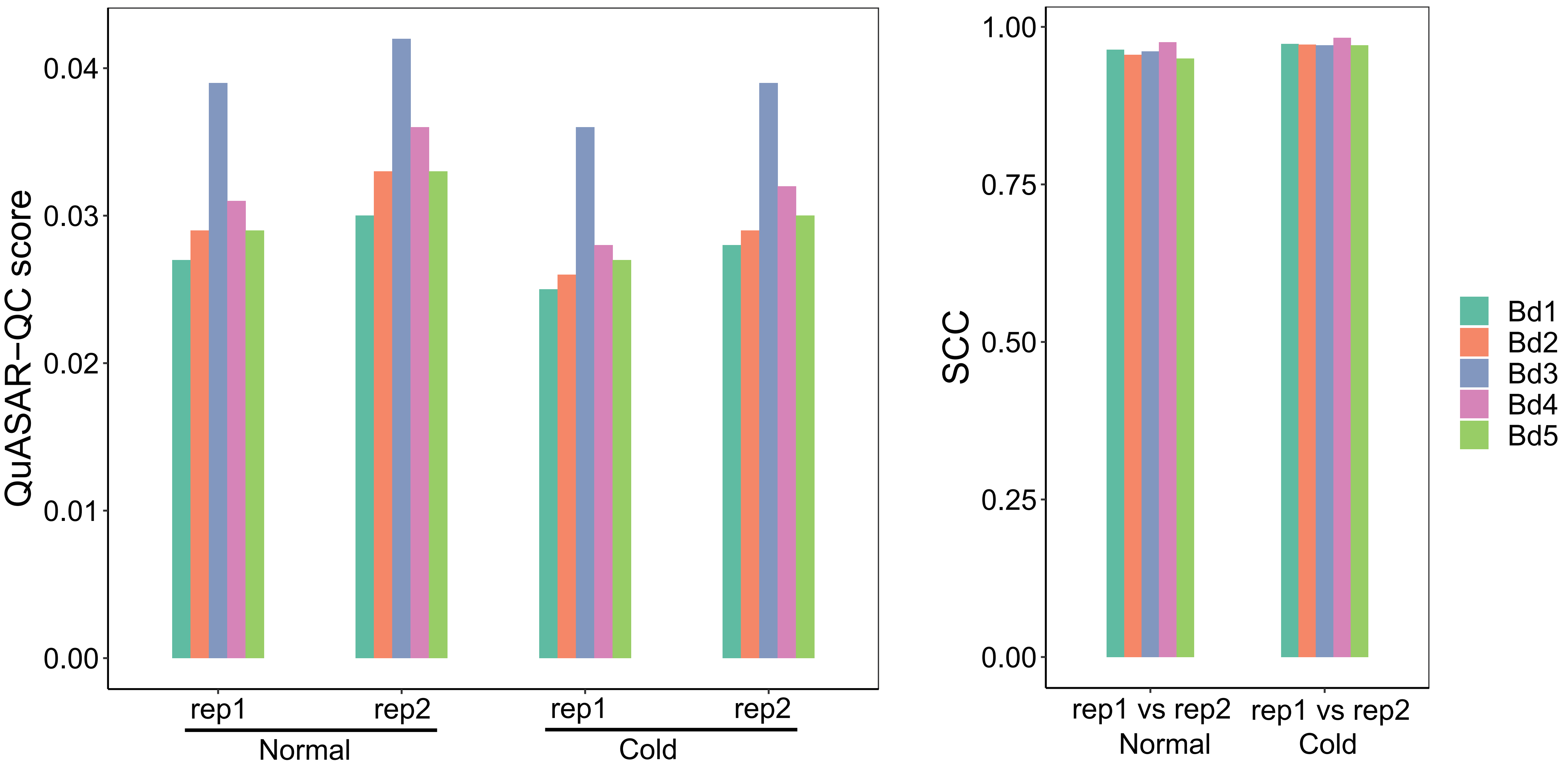

Figure S1. Quality measures for the Hi-C maps.
QuASAR-QC score (left panel) and stratum-adjusted correlation coefficient (SCC) (right panel) were calculated using 3DChromatin_ReplicateQC toolkit at 50 kb resolution for the Hi-C maps.

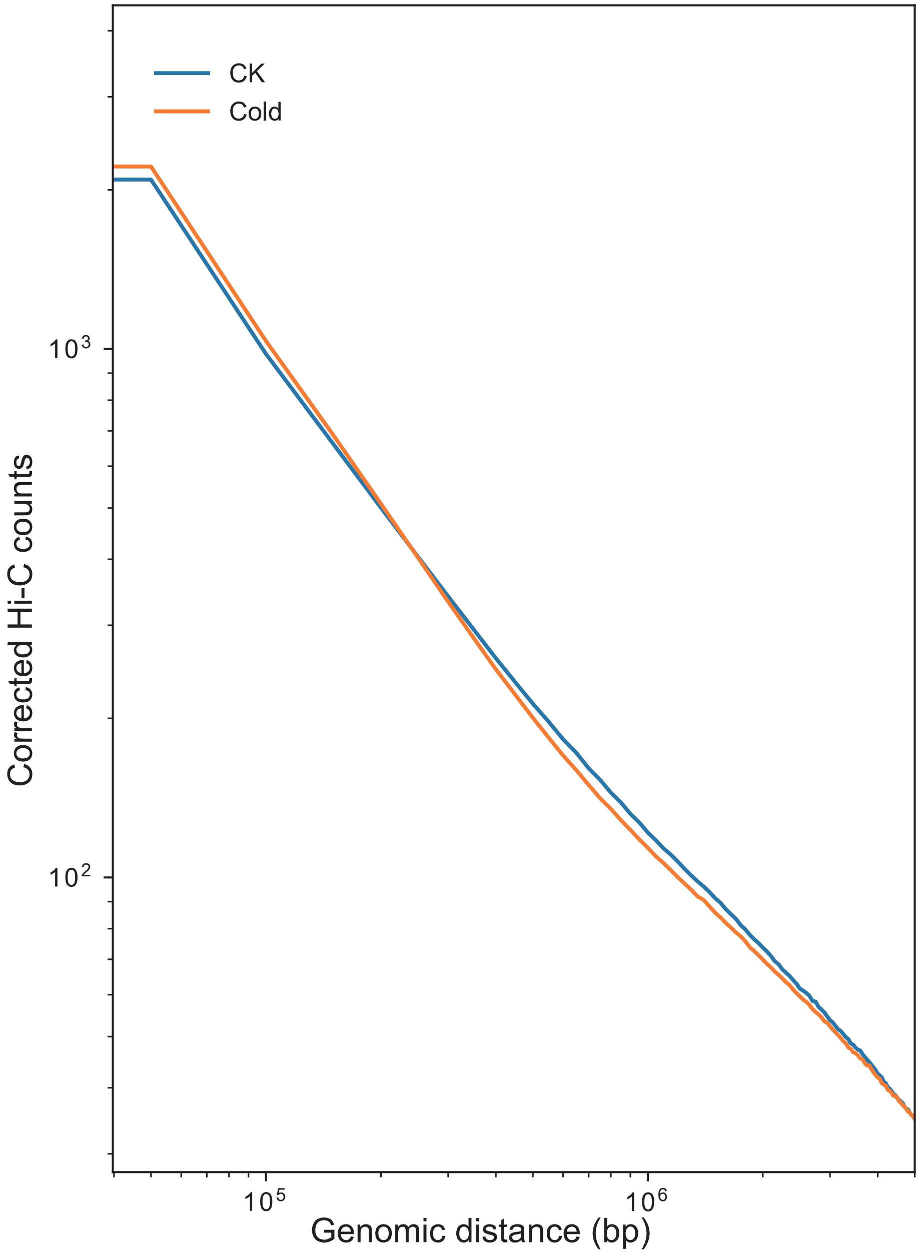

Figure S2. Interaction decay exponents along with genomic distance at 50-kb resolution of normal (blue) and cold-treated (red) Bd21.

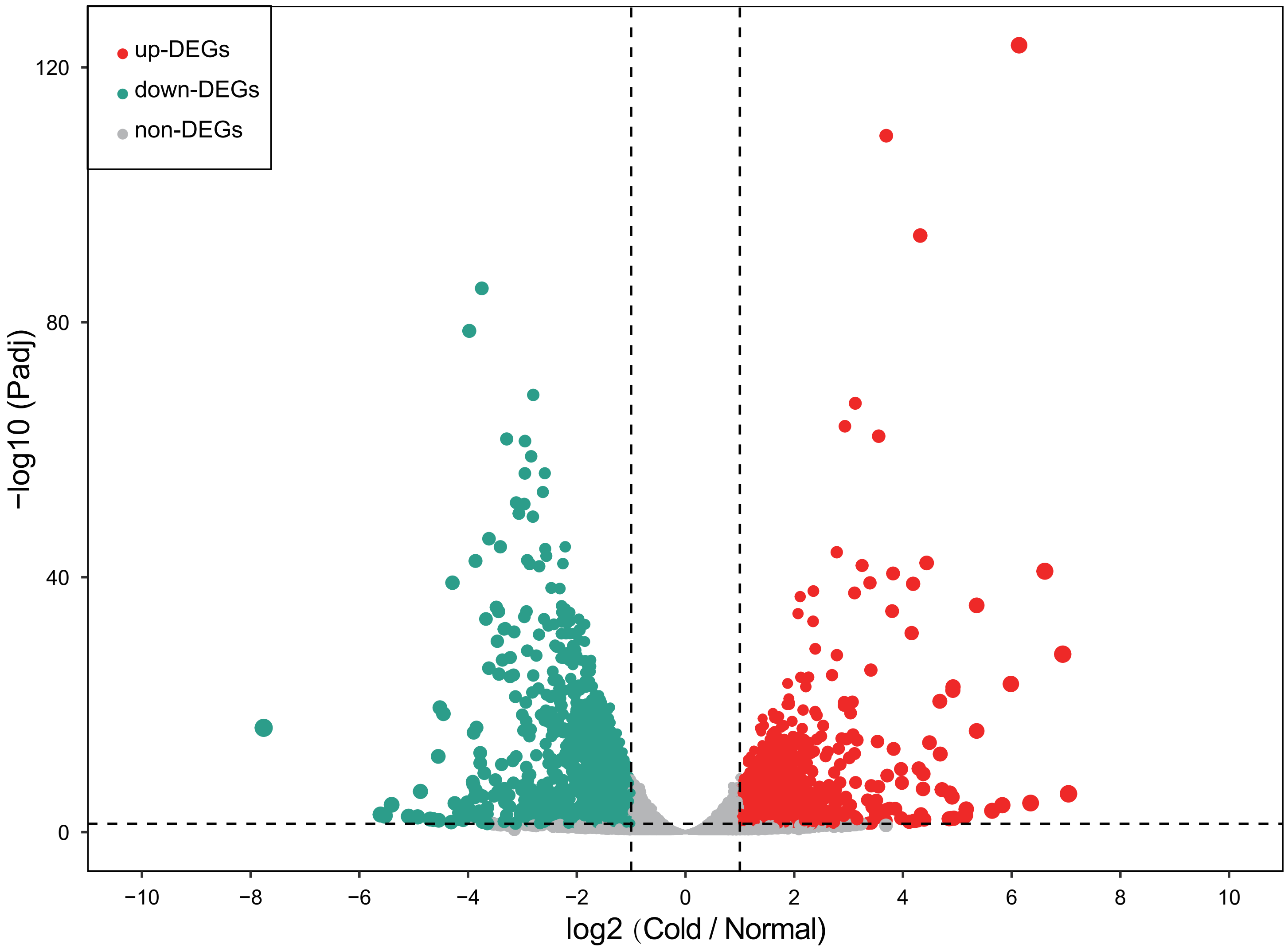

Figure S3. Volcano plots of differentially expressed genes (DEGs) in Cold vs. Normal samples.
Red points refer to up-regulated DEGs and blue points refer to down-regulated DEGs, whereas grey points indicate non-DEGs.

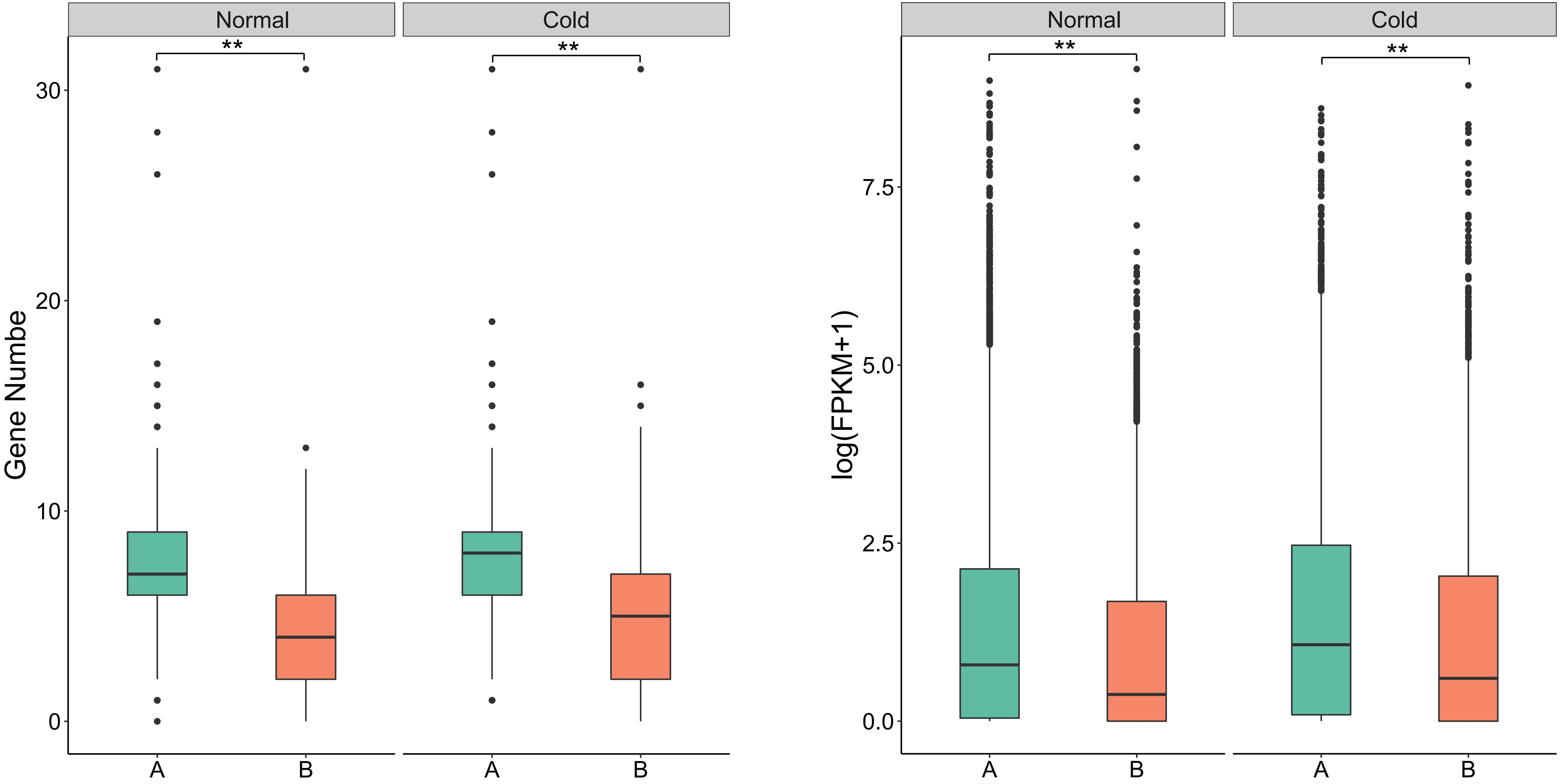

Figure S4. Illustration of gene density and gene expression (FPKM value) of A compartments and B compartments in the normal and the cold-treated Bd21, respectively.

The Wilcoxon test was used to analyze significance. '**' represents a P value < 0.01.

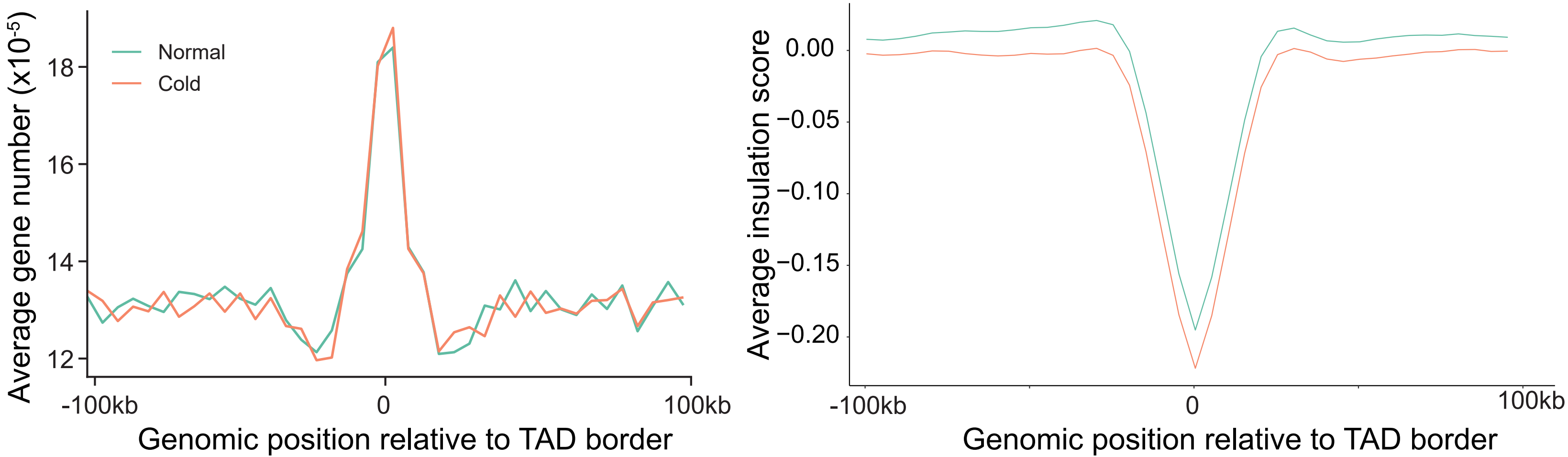

Figure S5. Genes enrichment and insulation score profiles around TAD boundaries at the two conditions.

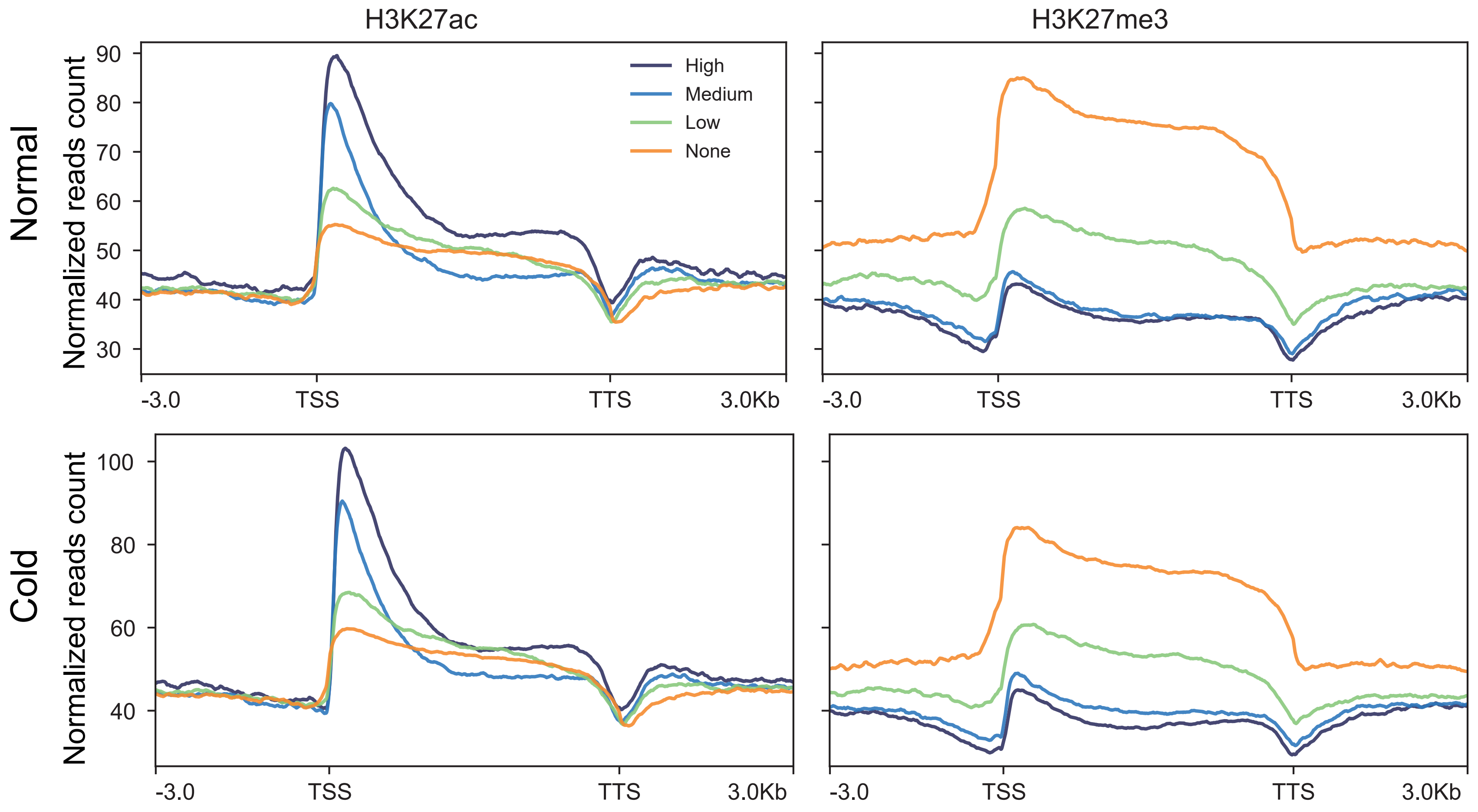

Figure S6. Distribution of histone modifications (H3K27ac and H3K27me3) surrounding genes in the normal and the cold-treated Bd21, respectively.
Genes were grouped based on expression levels. Expressed genes with FPKM values greater than 0.1 were equally divided into 3 groups according to expression levels (high, medium and low).

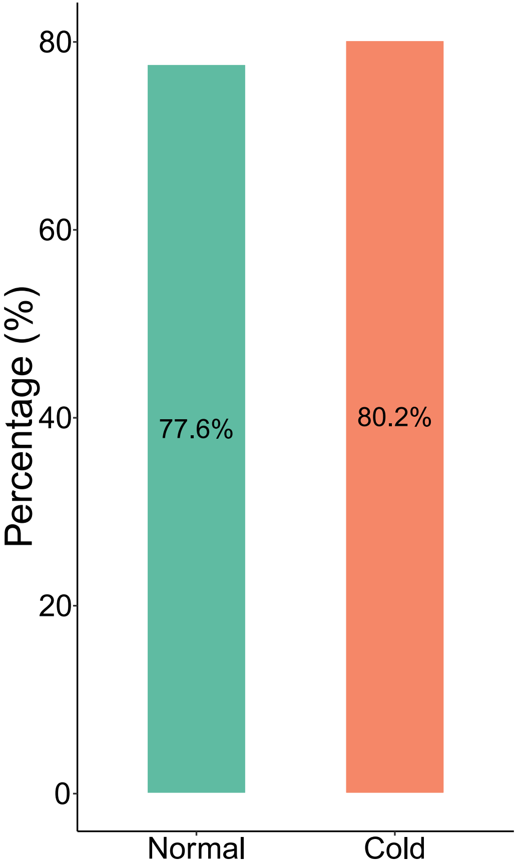

Figure S7. The proportion of loop anchors overlapped with DNase-seq peaks (DHSs).

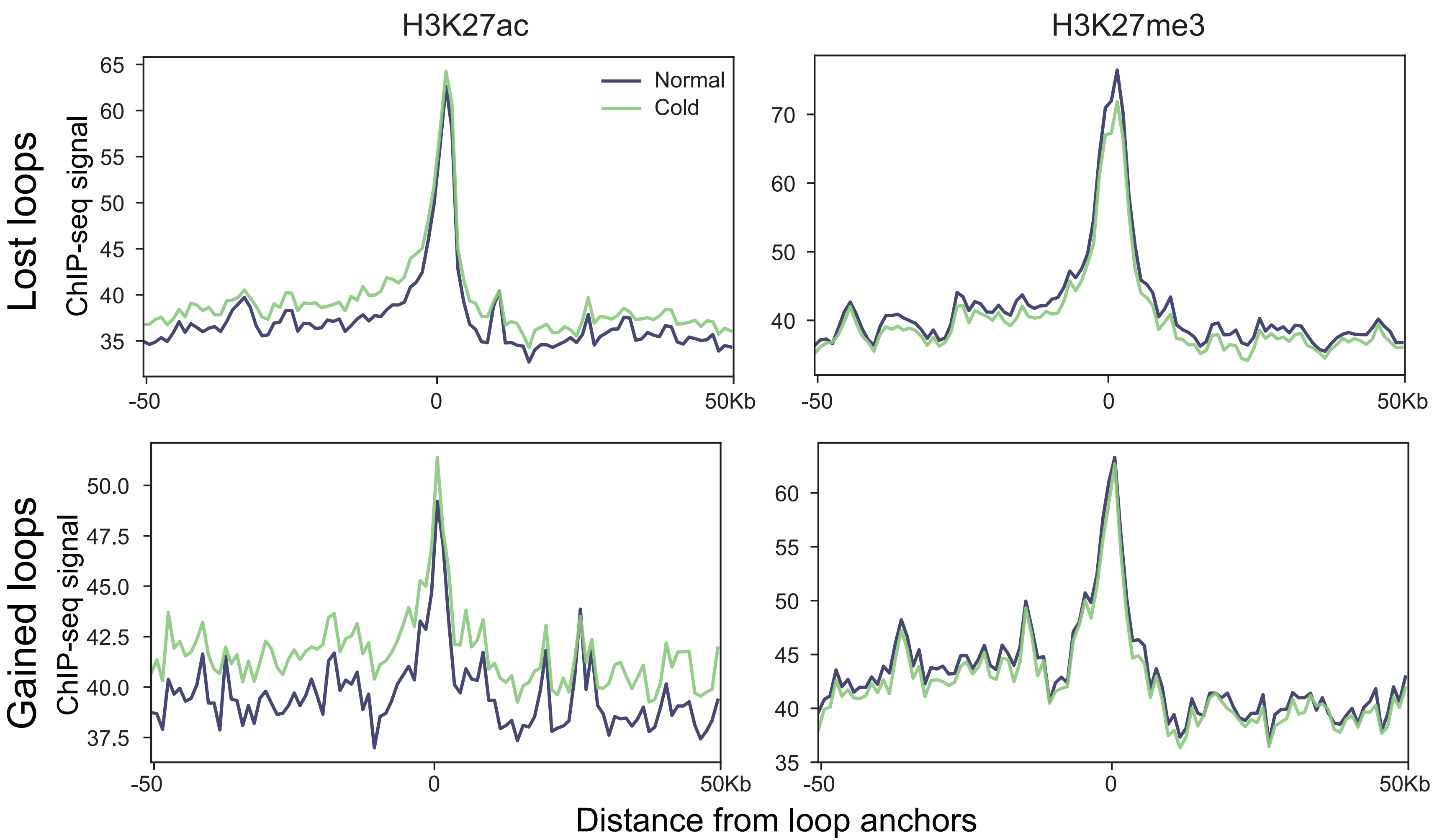

Figure S8. Line plots showing the normalized tag intensity of H3K27ac (left) or H3K27me3 (right) data from normal or cold-treated Bd21 at lost or gained loop anchors.

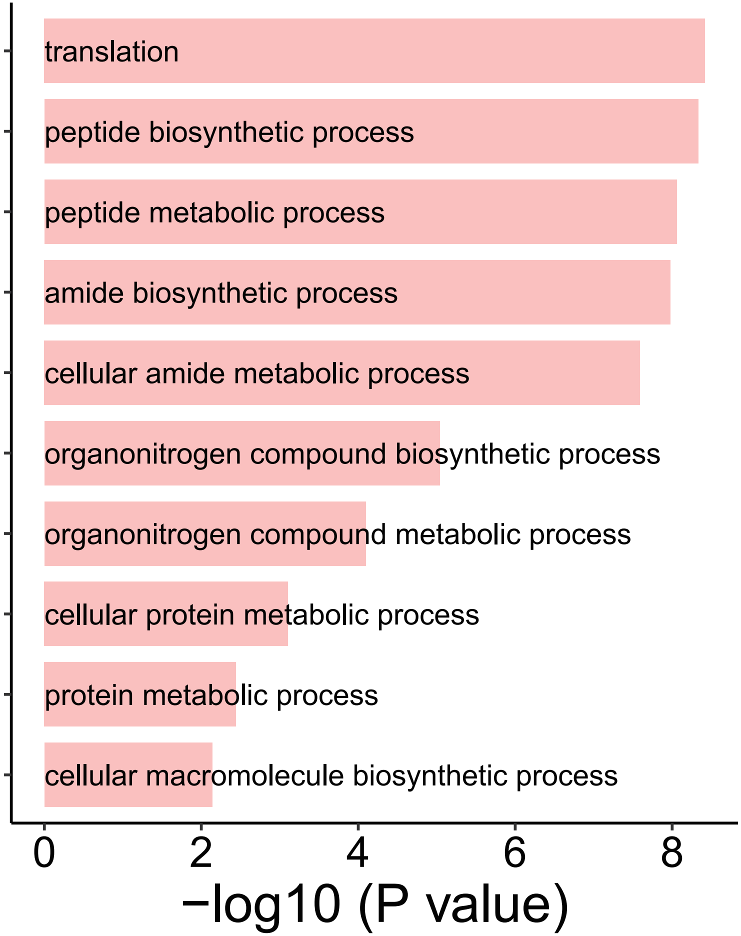

Figure S9. GO biological process analysis of DEG genes associated with genome reorganization.
The top ten enriched GO biological processes are indicated.
